# Surpassing Shockley–Queisser Efficiency Limit in Photovoltaic Cells

**DOI:** 10.1007/s40820-025-01844-8

**Published:** 2025-07-14

**Authors:** Zhigang Li, Bingqing Wei

**Affiliations:** 1 School of Materials Science and Engineering, Taizhou University, Taizhou, 318000 People’s Republic of China; 2 Department of Mechanical Engineering, University of Delaware, Newark, DE 19716 USA

**Keywords:** Single-junction Si solar cells, Power conversion efficiency, Shockley–Queisser model, Carrier freeze-out effect

## Abstract

The Shockley–Queisser (S-Q) model sets a theoretical limit on the power conversion efficiency (PCE) of single-junction solar cells at around 33%. Recently, a PCE of 50%-60% was achieved for the first time in n-type single-junction Si solar cells by inhibiting light conversion to heat at low temperatures. Understanding these new observations opens tremendous opportunities for designing solar cells with even higher PCE to provide efficient and powerful energy sources for cryogenic devices and outer and deep space explorations.

## Highlights

- A record power conversion efficiency of 50%–60% was achieved in Si solar cells by inhibiting the lattice atoms’ thermal oscillations at low temperatures.
- Enhancing the light penetration depth can effectively mitigate carrier freeze-out and expand the operational temperature range of silicon cells to 10 K.

## Graphical abstract

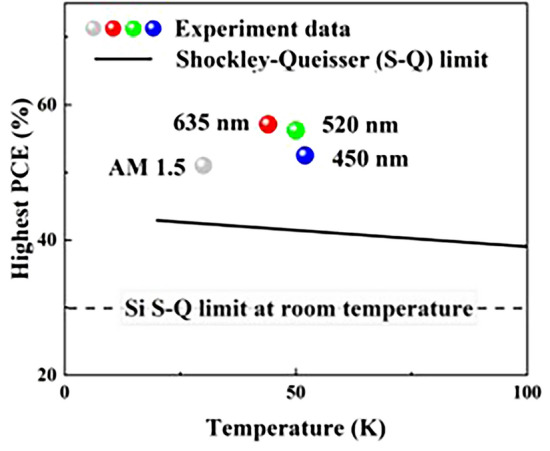

## Introduction

The Shockley–Queisser (S-Q) model is widely recognized in the scientific community, setting a theoretical limit on the power conversion efficiency (PCE) of single-junction solar cells at around 33% [1]. This limitation arises because most solar energy is lost as heat, especially from photons with energies significantly higher than the solar cell’s bandgap. Breaking the S-Q limit to achieve a considerably higher PCE is highly desirable and a long-standing pursuit for both fundamental and applied research.

To overcome this efficiency barrier, two primary strategies have been proposed [1]. The first involves extracting hot carriers before they lose energy and settle at the band edge, potentially enabling the generation of an open-circuit voltage (V_OC_) higher than the bandgap (E_g_). The second strategy focuses on increasing photocurrent by generating multiple electron–hole pairs through impact ionization, which could lead to an external quantum efficiency (EQE) greater than 100%. Despite these promising approaches, surpassing the S-Q efficiency limits in solar cells remains a formidable challenge.

Reducing the temperature to inhibit thermal loss is a straightforward method; extensive research has examined the impact of temperature on the PCE of solar cells [1]. However, when the temperature is < 150–200 K, the PCE will decrease with decreasing temperature due to the effects linked to carriers. The hypothesis that increasing PCE by cooling no longer applies at low temperatures appears to challenge the law of thermodynamics. Furthermore, the temperature limitation constrains their utility for cryogenic apparatuses and exploratory missions in outer, deep, or planetary environments, such as the lunar South Pole, where the temperature hovers around 30–50 K [2].

## Classical Solar Cell Theory is No Longer Applicable at Ultra-Low Temperatures

According to traditional theory^1^, thermal loss correlates with the non-equilibrium dynamics of photocarriers excited by photons with energies much higher than the band gap. This is followed by a rapid cooling process, which extends downward to the band edges, releasing excess energy in the form of phonons. As well known, phonon numbers embody the lattice atoms’ thermal oscillations within a solid crystal and depend on the temperature. However, the traditional theory may face challenges when applied to solar cells operated at extremely low temperatures. For instance, what will happen if the number of phonons the solar cells can hold is close to or less than that of the released phonons due to the relaxation of photocarriers back to the bandgap’s edge? Where does the suppressed thermal loss energy go if the traditional theory about the phonon-releasing process is inapplicable? How would it impact the PCE?

**Fig. 1:**
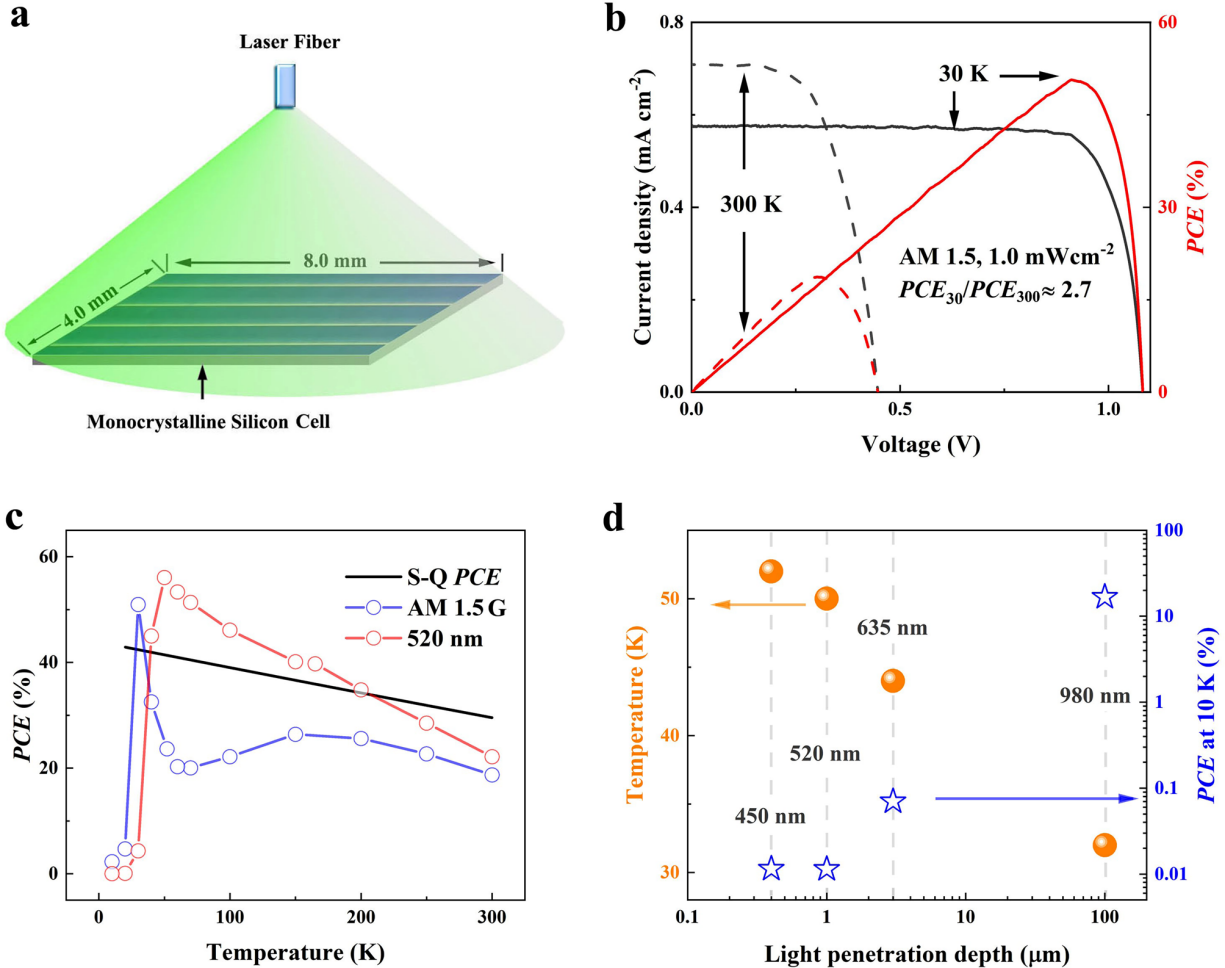
**a** Schematics of the experimental setup for photoelectric property measurements. **b** J–V/P–V solid/dash curves at 300 and 30 K. Here, PCE50 and PCE300 denote the PCE of the cell at 30 and 300 K, respectively. **c** Temperature-dependent *PCE* experimental data and calculation curve based on the S-Q theory (black line). **d** Effect of light penetration depth on the highest output power temperature (orange dots) and the PCE at 10 K (blue stars). The light penetration depths at 450/520/635/980 nm are approximately 0.4/1.0/3.0/100 μm, respectively [2]. Copyright 2024, John Wiley and Sons

A recent breakthrough has demonstrated that thermal losses in monocrystalline single-junction silicon solar cells—the most commonly used type—can be effectively reduced by enhancing carrier mobility through temperature regulation [2]. Using homochromatic lasers with different photon energies ΔE ($\hbar \omega - E_{g}$, where $\hbar \varpi$ represents the photon energy), researchers were able to double the PCE of these solar cells to 50%–60% at extremely low temperatures (30–50 K, Fig. 1a-c). At 30 K, the PCE under AM 1.5G illumination reached about 51%, nearly double the record efficiency of 27.3% achieved at room temperature [3], and approximately 20% higher than the S-Q limit at the same temperatures (Fig. 1c). It is exciting and necessary to discuss the determinants of the ultrahigh PCE from new perspectives.

## Temperature-Dependent Photoelectric Characteristics

V_OC_ is a critical factor in solar cell performance, as a higher V_OC_ typically leads to a higher PCE. To increase photovoltage, the transport time of photocarriers through the lattice matrix and their extraction at the electron/hole transfer layer must be faster, indicating a higher carrier mobility. In other words, higher carrier mobility results in a higher V_OC_. Theoretically, if the transport time is shorter than the cooling time of hot carriers, the V_OC_ can exceed the energy bandgap E_g_ [1].

Experimental results demonstrated a strong correlation between V_OC_ and carrier mobility [2]. As the temperature decreases, the V_OC_ increases and eventually approaches approximately 1.1 V, which is close to the bandgap of silicon (E_g_ ≈ 1.12 V), while the carrier mobility rises from approximately 1.1 × 10^3^ to 4.6 × 10^4^ cm^2^ V^−1^ s^−1^. This suggests a strong correlation between these two properties in the studied material system, with both exhibiting monotonic increases as the temperature decreases. Moreover, this behavior is so robust and independent of light intensity.

A hot electron–hole pair can be split into two or more pairs via impact ionization, leading to an EQE greater than 100% [1]. This process is typically initiated by photons with energy greater than 2.25 times the material’s bandgap (E_g_) at room temperature. By confining the hot carriers spatially and quantizing their energy levels, it is possible to relax the momentum conservation rules and amplify many-body interactions among the carriers. This can enhance the efficiency of multiple exciton generation (MEG), where more than one electron–hole pair is produced per absorbed photon.

Under AM 1.5G illumination, an unusual rise in J_SC_ has been observed, increasing from 0.41 mA cm^−2^ at 60 K to 0.58 mA cm^−2^ at 30 K^2^. This behavior resembles the MEG effect. However, unlike at room temperature, low-energy photons, such as those at 980 nm (~ 1.13 E_g_), can trigger MEG at extremely low temperatures. This suggests that at such low temperatures, MEG is initiated when the photocarrier energy (ΔE) surpasses the freeze-out energy of carriers (i.e., k_B_ΔT, ~ 0.01 eV, k_B_ is Boltzmann constant) rather than the material’s bandgap. By reducing thermal losses of solar cells at these temperatures, photocurrent can increase, potentially offering a pathway to surpassing the S-Q efficiency limit.

## Overcome the Freeze-Out Effect at Low Temperatures

A key characteristic of semiconductors at very low temperatures is the carrier freeze-out effect, which is caused by partially ionized impurities or defects in the material. The charge carriers based on thermal excitation become “trapped” or “frozen” at the impurity or defect energy levels as the temperature drops. The freeze-out effect is highly temperature-dependent, resulting in a nearly zero PCE at extremely low temperatures.

Unlike thermally excited charge carriers, photocarriers are unaffected by the freeze-out effect and can theoretically exist even at zero kelvin as long as photons are present. The photocarrier density depends on the cell’s thickness and light penetration. Experiments show that J_SC_ is mainly determined by the photocarriers through the bottom of solar cells. Enhancing the light penetration depth, from nanometers to microns, can effectively mitigate the freeze-out effect (Fig. 1d) and improve the PCE of Si cells at extremely low temperatures [4].

Theoretically, near zero kelvin, the number of phonons is nearly zero, meaning thermal losses could be entirely suppressed, allowing PCE to approach the light absorptivity of the solar cell. However, the carrier freeze-out effect can reduce J_SC_ to be negligible at such low temperatures, leading to a sharp decline in PCE. The interplay between these opposing effects—reduced thermal losses and carrier freeze-out—creates the potential for a peak in PCE at low temperatures.

The carrier freeze-out effect can cause the J_SC_ to approach zero, resulting in a PCE decrease close to zero. The freeze-out effect is strongly dependent on temperature; the lower the temperature, the stronger the effect. The highest PCE temperatures shift from 52 to 44 K, corresponding to a change in wavelength from 450 to 635 nm (Fig. 1d). This shift is attributed to the increased light penetration depth necessary to overcome the freeze-out effect.

Unlike free charge carriers, the photocarriers are unaffected by the freeze-out effect and hypothetically survive even at zero kelvin when photons are available. Due to the lack of free carriers at low temperatures (the carrier freeze-out effect), the behavior of the Si cell primarily relies only on the intensity of the photocarriers. As these carriers traverse downward through the thickness, their density gradually diminishes due to the reduced photoelectric effect, resulting in a decrease in photocarrier intensity by the time they reach the bottom of the thickness. The J_SC_ of the Si cell is determined by the maximum value allowed through the thickness of the cell, which depends on its photocarrier density. For a specific thickness of Si cells, the photocarrier density of the bottom layer is determined by the light intensity reaching it. Hence, the PCE of Si cells can be adjusted to effectively overcome the freeze-out effect by increasing the light penetration depth and/or decreasing the cell thickness to micro- or even nanometer scale at low temperatures.

## Applications and Challenges

There are two main approaches to enhancing the efficiency of solar cells at low temperatures. The first is to minimize unnecessary energy losses, such as light and heat losses. To reduce light energy loss, broadband and omnidirectional subwavelength antireflective films can significantly minimize reflection, while tandem multi-junction designs [4] and photon recycling architectures effectively reduce transmission losses. Lowering the temperature helps reduce heat loss but exacerbates the carrier freeze-out effect, which can severely impact performance.

Another approach to enhancing efficiency is to increase the bottom photocarrier density at low temperatures. This can be achieved by reducing the thickness of the solar cell or using light waves with greater penetration depth. However, both methods may lead to increased light transmission losses. A potential solution lies in combining multi-junction designs and photon recycling, carefully selecting laser wavelengths, and optimizing cell thickness to achieve optimal performance. This combination could allow tuning the temperature at which peak output power occurs to a lower level, potentially approaching near zero kelvin, thereby achieving a higher PCE.

V_OC_ and J_SC_ are vital parameters in determining solar cell efficiency. High-mobility materials allow for high-V_OC_ solar cells due to the correlation between V_OC_ and carrier mobility. High carrier density (heavily doped) materials can lead to solar cells with high J_SC_. However, increasing carrier density in a material generally reduces its mobility, making it challenging to achieve both high carrier density and mobility simultaneously. Certain high-mobility materials, such as graphene and topological materials [5], have been identified as promising candidates for combining high carrier mobility and density, potentially enabling the development of ultrahigh-efficiency solar cells at room temperature.

Specific solar cell designs that minimize energy losses can further enhance PCE. For example, concentration solar cells can harness the thermoelectric effect to reduce thermal losses, improving overall efficiency. However, this approach falls outside the scope of the current discussion.

Although ultrahigh PCEs surpassing the S-Q limit have been achieved in single-junction silicon solar cells, several fundamental and practical questions remain. First, what is the theoretical limit of PCE in the absence of thermal losses? Second, what is the optimal thickness/structure for single- or multi-junction Si solar cells when exposed to different wavelength lasers or full solar spectrum in practical application? Furthermore, can ultrahigh PCEs be realized in other types of solar cells, such as GaAs or perovskites? If not, where does the excess energy dissipate?

Furthermore, the space environment differs markedly from that of ground-based laboratories. The extreme conditions in deep space, including wide temperature fluctuations, intense cosmic radiation, and dust accumulation, significantly affect the performance and longevity of solar cells. A systematic evaluation of the effects of rapid thermal cycling (ranging from cryogenic to high temperatures) and prolonged exposure to high-energy cosmic radiation is highly desirable. To ensure reliable PCE under such harsh conditions, specialized experimental protocols, structural optimizations, and stress-performance modeling are essential. In addition, the long-term stability of silicon solar cells under ultra-low-temperature conditions warrants thorough investigation.

The ultrahigh PCE achieved at extremely low temperatures opens up a novel approach to using light as an efficient wireless power source for space and planetary exploration devices. For example, in the perpetually shadowed craters at the Moon’s South Pole, where temperatures range from 30 to 50 K and vast amounts of water are present, reflective or light-emitting devices could be placed in sunlit areas to provide remote wireless charging for mining robots equipped with solar panels and operating in shadowed regions. In addition, an ultrahigh PCE at extremely low temperatures, i.e., cosmic background radiation temperatures (~ 2.7 K), would enable outer space probes to receive a constant supply of energy through starlight, thereby facilitating the acceleration of exploration of the deeper universe.

## References

[1] K. Wang, L. Zheng, Y. Hou, A. Nozariasbmarz, B. Poudel et al., Overcoming Shockley-Queisser limit using halide perovskite platform? Joule **6**(4), 756–771 (2022). 10.1016/j.joule.2022.01.009

[2] Z. Li, Y. Chen, R. Guo, S. Wang, W. Wang et al., Doubling power conversion efficiency of Si solar cells. Adv. Mater. **36**(48), 2405724 (2024). 10.1002/adma.202405724 10.1002/adma.202405724 39188194

[3] M.A. Green, E.D. Dunlop, M. Yoshita, N. Kopidakis, K. Bothe et al., Solar cell efficiency tables (version 63). Prog. Photovolt. Res. Appl. **32**(1), 3–13 (2024). 10.1002/pip.3750

[4] Z. Wang, L. Zeng, T. Zhu, H. Chen, B. Chen et al., Suppressed phase segregation for triple-junction perovskite solar cells. Nature **618**(7963), 74–79 (2023). 10.1038/s41586-023-06006-7 36977463 10.1038/s41586-023-06006-7

[5] Z. Lu, T. Han, Y. Yao, A.P. Reddy, J. Yang et al., Fractional quantum anomalous Hall effect in multilayer graphene. Nature **626**(8000), 759–764 (2024). 10.1038/s41586-023-07010-7 38383622 10.1038/s41586-023-07010-7

